# Physiological occlusal force attenuates replacement root resorption of replanted teeth: an experimental animal study

**DOI:** 10.1186/s12903-024-04394-4

**Published:** 2024-06-05

**Authors:** Zhenjiang Ding, Anqi Wang, Yao Liu, Shu Zhu, Liming Jiang, Xu Chen

**Affiliations:** 1 Department of Paediatric Dentistry, School and Hospital of Stomatology, China Medical University, Shenyang, China; 2 Liaoning Provincial Key Laboratory of Oral Diseases, Shenyang, China; 3 Shanghai Stomatological Hospital and School of Stomatology, Fudan University, Shanghai, China; 4 Shanghai Key Laboratory of Craniomaxillofacial Development and Diseases, Shanghai, China

**Keywords:** Physiological occlusal force, Replanted teeth, Replacement root resorption, Osteoclasts

## Abstract

**Background:**

Tooth avulsion represents the most severe form of dental trauma, necessitating tooth replantation as the primary treatment. However, the risk of replacement root resorption (RRR) poses a significant threat to tooth retention following replantation. This study preliminarily aimed to investigate the effect of physiological occlusal force on RRR after the replantation of avulsed teeth and to explore the potential underlying mechanisms.

**Methods:**

Thirty-six 4-week-old male Sprague-Dawley rats underwent extraction and immediate replantation of their left maxillary molars. The rats were randomly divided into two major groups: the occluded (*n* = 18) group, where the opposite mandibular teeth were preserved; non-occluded (*n* = 18) group, where the opposite mandibular teeth were extracted. Within each major group, there were three subgroups corresponding to 7 days, 14 days, and 2 months, resulting in a total of six subgroups, (*n* = 6 per subgroup). The right maxillary first molars served as the normal control. Various periodontal characteristics were assessed using haematoxylin-eosin (H&E), tartrate-resistant acid phosphatase (TRAP) staining, and micro-computed tomography (micro-CT).

**Results:**

Histological staining revealed that under occlusal force, the early stage (day 7) after tooth replantation mainly manifested as root surface resorption, especially in the non-occluded group, which gradually diminished over time. Cementum and periodontal ligament (PDL) repair was observed on day 14. Micro-CT analysis indicated a significant decrease in PDL width in the non-occluded group two months after replantation, consistent with the histological findings, signifying severe RRR in the non-occluded group.

**Conclusions:**

This study provides preliminary evidence that physiological occlusal force may attenuate osteoclastogenesis during the early stage of tooth replantation, thereby reducing the occurrence of RRR and promoting periodontal healing.

## Background

Tooth avulsion, representing a severe form of dental trauma, accounts for 0.5–16% of all dental trauma cases [1]. Immediate and appropriate replantation of the avulsed tooth is crucial. The success of tooth replantation is contingent upon several factors, including the condition of the periodontal ligament (PDL), the stage of root development, and the duration the tooth remains outside the oral cavity [2]. However, a significant complication associated with tooth replantation is replacement root resorption (RRR), also known as dentoalveolar ankylosis. This process typically begins with PDL necrosis, often resulting from improper tooth preservation or prolonged extraoral time. It involves the progressive replacement of the tooth root by bone tissue, leading to the eventual loss of the replanted tooth [3]. The International Association of Dental Traumatology (IADT) recommends immediate replantation at the accident site to reduce extra-alveolar time, which is crucial for preserving PDL cell viability and preventing RRR. If immediate replantation is unfeasible, it is recommended to store the avulsed tooth in appropriate media such as milk, Hank’s balanced salt solution, saliva, or saline until professional care is accessible. Post-replantation care involves careful examination and stabilisation of the tooth with a passive flexible splint for up to 2 weeks, supporting initial PDL healing. Systemic antibiotics are recommended to prevent infection and endodontic treatment within 2 weeks for mature teeth with closed apices [4]. Despite these guidelines, 73–96% of replanted teeth are still lost [5], indicating the challenging prognosis of dental avulsion.

The PDL, a dense fibrous connective tissue located between the tooth root and alveolar bone, plays a vital role in supporting teeth and responding to mechanical forces, such as those experienced during mastication [6, 7]. Physiological occlusal force, as a natural component of mastication, is essential for maintaining the health and integrity of the PDL. In the absence of such force, atrophic changes in the PDL including narrowing of the PDL space and misalignment of collagen fibres may occur [8]. Conversely, the presence of physiological occlusal force is hypothesised to promote the reparative healing of the injured PDL, a concept supported by clinical guidelines that recommend the use of flexible splints for replanted teeth to maintain normal occlusal force during the healing process [4, 9].

Considering the critical role that physiological occlusal force may play in maintaining PDL health, this study preliminarily aimed to investigate the potential mitigating effects of physiological occlusal force on RRR following tooth replantation, proposing an initial hypothesis that physiological occlusal force post-replantation could help in reducing the incidence of RRR. This experimental animal study aimed to initiate exploration of potential mechanisms through which occlusal force influences PDL healing and RRR progression.

Furthermore, this study is contextualized within the broader scope of bone tissue homeostasis. Mechanical forces are known to influence the behaviour of osteoblasts and osteoclasts (OCs), which are crucial in bone remodelling [10, 11]. Root resorption involves processes similar to bone resorption, where the balance between osteoclast-induced bone resorption and osteoblast-mediated bone formation is disrupted [12]. Our research aimed to preliminarily explore the mechanisms underlying the modulation of this balance by physiological occlusal forces, potentially influencing the prevention of RRR in replanted teeth.

## Methods

### Rats and tooth replantation

Our controlled experimental animal study was conducted over a two-month period at the Laboratory Animal Centre of China Medical University. A total of 36 4-week-old male Sprague-Dawley rats weighing 110 ± 10 g were acquired from SPF (Beijing) Biotechnology Co., Ltd. (Beijing, China). The inclusion criteria for the study were good health status with normal feeding, while exclusion criteria encompassed signs of illness, infection, abnormal behaviours, or dental or maxillofacial deformities.

The in vivo experiments complied with the ARRIVE guidelines and were approved by the Institutional Animal Care and Use Committee of China Medical University (Approval No. 2,020,194). Rats were housed in a controlled 12-h light/dark cycle at a constant temperature of 25℃, ensuring a consistent environment for the duration of the study.

Rats were systematically randomised into two principal groups: the occluded (*n* = 18) group and the non-occluded (*n* = 18) group. Sample size analysis was conducted using G*Power software (latest ver. 3.1.9.7; Heinrich-Heine-Universität Düsseldorf, Düsseldorf, Germany) with a statistical power of 80%, a significance level of 0.05, and an effect size of 0.9. A minimum of 16 samples per group was determined as the sample size. Each group was further divided into three subgroups based on the observation period (7 days, 14 days, and 2 months), resulting in six subgroups (*n* = 6 per subgroup). This random allocation employed a computer-generated sequence to ensure equal distribution across the groups. In the non-occluded group, the left mandibular first molars were extracted, while in the occluded group, they were left untreated. The right maxillary first molars were designated as normal controls, with the mesial root of the first molar in the maxilla chosen for observation.

For the surgical procedures, rats were anaesthetised with an intraperitoneal injection of 40 mg/kg of 1% pentobarbital sodium solution (Merck, Germany). The left maxillary first molar was selected as the experimental tooth, and a minimally invasive tooth extraction was performed. Initially, a dental probe was used to separate the gingival tissue precisely. A #5 needle was then utilized to delicately mobilise the tooth, which was carefully extracted using a needle holder. Haemostasis was achieved using sterile, dry cotton balls. Simultaneously, the tooth was promptly immersed in physiological saline and replanted in its original position after a 5-minute interval. Post-operative care included a soft diet and a 3-day course of 20,000 IU/day penicillin sodium (Shenyang Pharmaceutical, China).

Throughout the study, we meticulously monitored the rats for any signs of post-replantation discomfort, infection, significant weight variations, or dental complications such as tooth loss, periodontal disease, or other dental abnormalities affecting the integrity of the replantation site. Any rats exhibiting these conditions were carefully assessed and excluded when necessary to ensure their well-being and data reliability. The investigators responsible for the surgical procedures, care, and data collection were blinded to the group assignments, thus maintaining the study’s unbiased nature and scientific validity.

### Histological preparation and haematoxylin-eosin (H&E) staining

The rats were euthanised 7 days, 14 days, and 2 months respectively after replantation, and then tissues were collected immediately. All specimens were fixed in 4% paraformaldehyde solution (Boster, China) and decalcified with 10% ethylenediaminetetraacetic acid solution (Boster, China) for two months. Subsequently, the specimens were embedded in paraffin, sectioned parallel to the long root axis of the mesial root of the left maxillary first molar at a thickness of 3.5 μm, and then stained with H&E. The sections were observed and photographed under a microscope (ECLIPSE 80i, Nikon, Japan). The histopathological periodontal healing mode of replanted teeth was evaluated according to the standards listed in Table 1.

**Table 1:** Histopathologic periodontal healing mode of replanted teeth

Healing types	Histopathology
PDL healing	PDL is completely regenerated without resorption lacunae and inflammatory cell infiltration [13].
Surface resorption	There is no inflammatory cell infiltration, resorption lacunae or new cementum appear on the root surface to repair the locally resorbed area on the root surface and the reattachment of PDL fibres [14].
Inflammatory resorption	Resorption lacunae appear in cementum and/or dentine, and adjacent periodontal tissues are infiltrated by inflammatory cells such as lymphocytes and plasma cells [15].
RRR	The PDL space disappears, the surface of the alveolar bone and root fuses, and the absence of periodontal ligament cells (PDLCs) on the root surface [16].

### Tartrate-resistant acid phosphatase (TRAP) staining

TRAP staining was performed using a TRAP staining kit (Besibio, China), according to the manufacturer’s instructions. This evaluation was carried out exclusively on the groups sacrificed at 7 days and 14 days post-replantation. TRAP-positive multinucleated cells along the root were quantified as OCs in 10 random areas of each specimen at 100× magnification (ECLIPSE 80i, Nikon, Japan).

### Micro-computed tomography (micro-CT) analysis

High-resolution micro-CT (SkyScan 1276, Bruker, Belgium) was used to investigate the changes in root morphology of the left maxillary first molars two months after replantation in both occluded and non-occluded groups. The right maxillary first molar was used as a normal control for comparison. The CT scanning parameters were set as follows: voltage, 85 kV; current, 200 µA; exposure time, 386 ms. The acquired CT images were reconstructed using the CT-Analyser software (version 1.17.9.0, Bruker micro-CT, Germany) and CT-Volume software (version 2.3.2.0, Bruker micro-CT, Germany) to generate three-dimensional reconstructions of the maxillae. Sagittal images of the mesial root of the maxillary first molar, displaying the maximum length, were obtained using the Data Viewer software (version 1.5.4.6, Bruker micro-CT, Germany). Additionally, horizontal plane images were captured at the mid-point of the root. To assess the width of the palatal PDL in this horizontal plane, the CT-Analyser software (version 1.17.9.0, Bruker micro-CT, Germany) was employed for measurement, and the average value was calculated based on previous research methodology [17].

### Statistical analysis

Experimental data are presented as the mean ± standard deviation. Comparisons between two groups were analysed using an independent two-tailed Student’s *t*-test, comparisons among more than two groups were performed using a one-way analysis of variance (ANOVA), and comparisons involving categorical data with small sample sizes were assessed using Fisher’s exact test with SPSS 23.0 (IBM Corp, Armonk, NY, USA). Statistical significance was set at *P* < 0.05.

Quantifying TRAP-positive cells and measuring PDL width were performed by two independent examiners. Intraclass correlation coefficient (ICC) was utilised to evaluate the interobserver reliability. The results revealed an ICC of 0.94 for TRAP-positive cells and 0.96 for PDL width measurement, indicating a high level of reliability between the examiners’ assessments (*P* < 0.05).

## Results

### Physiological occlusal force promoted PDL rearrangement of the replanted tooth at the early stage of the replantation

**Fig. 1:**
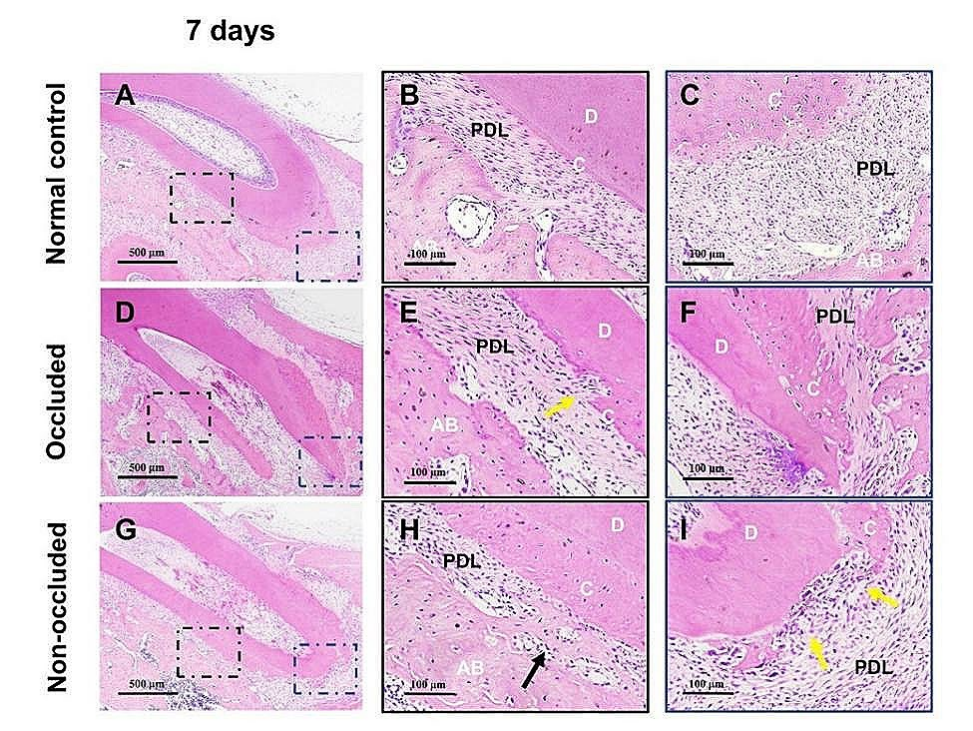
Histological observation at 7 days after replantation by H&E staining. A, The PDL fibres are well-arranged in the normal control group. B and C, High-magnification images of the middle and apical 1/3 of the root, respectively in (**A**). No root surface resorption is observed. D, The PDL fibres are irregularly arranged in the occluded group. E and F, Local magnification of the middle and apical 1/3 of the root, respectively in (**D**). A small number of absorption lacunae appear on the root surface (yellow arrowheads). G, The PDL fibres were irregularly arranged in the non-occluded group. H and I, Local magnification of the middle 1/3 and apical 1/3 of the root, respectively in (**G**). Some PDL spaces are replaced by bone/dentine-like tissue in the middle 1/3 area of the root (black arrowheads), and absorption lacunae are observed on the root surface, particularly in the apical 1/3 (yellow arrowheads). C, cementum; AB, alveolar bone; D, dentine; and PDL, periodontal ligament. The scale bars represent 500 μm (**A**, **D**, and **G**) and 100 μm (**B**, **C**, **E**, **F**, **H**, and **I**)

On post-replantation day 7, H&E staining revealed well-arranged PDL fibres with no observed resorption lacunae on the root surface in the normal control group (Fig. 1A-C). In the occluded group, the PDL fibres displayed an irregular arrangement, accompanied by a minor presence of resorption lacunae on the root surface (Fig. 1D-F). In the non-occluded group, PDL fibres were arranged irregularly, and more active resorption lacunae were observed on the root surface, especially in the apical third. Small osteoid tissues were observed between the roots and alveolar bone (Fig. 1G-I).

**Fig. 2:**
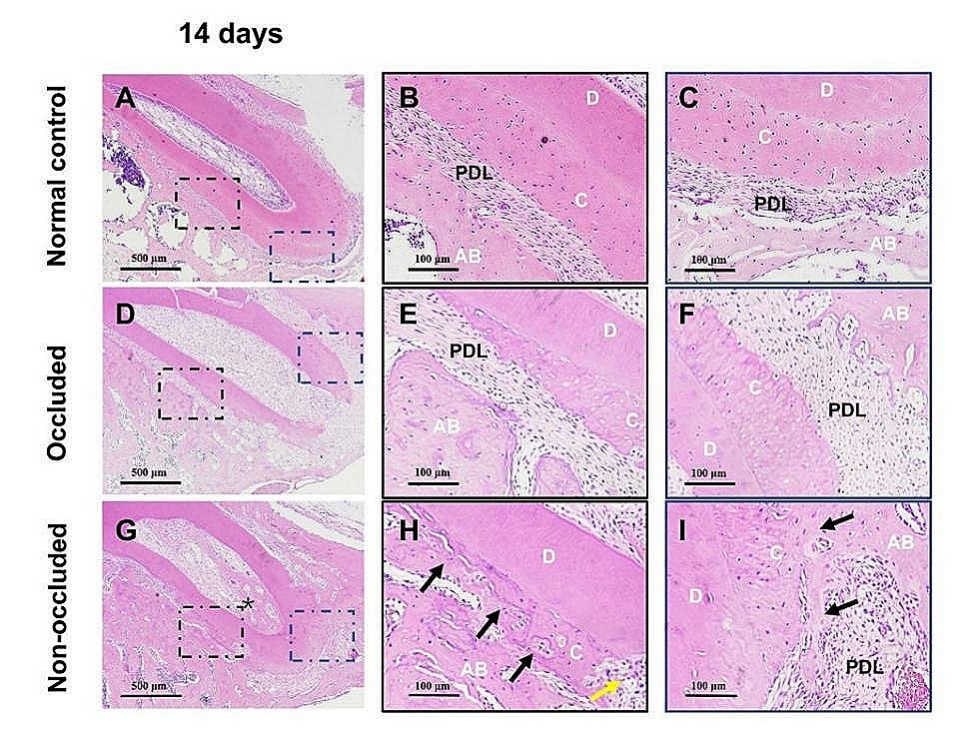
Histological observation 14 days after replantation by H&E staining. A, The PDL fibres are regularly arranged in the normal control group. B and C, High-magnification images of the middle and apical 1/3 of the root, respectively in (**A**). No root surface absorption is observed. D, The PDL fibres are rearranged regularly in the occluded group. E and F, Local magnification of the middle and apical 1/3 of the root, respectively in (**D**). A few small absorption lacunae appear on the root surfaces. G, The PDL space is significantly narrowed or even disappeared in the non-occluded group, and small bone/dentine-like tissue is observed in the root canal (black star). H and I, Local magnification of the middle 1/3 and apical 1/3 of the root, respectively in (**G**). Absorption lacunae are observed on the root surface (yellow arrowheads), and replacement root resorption (RRR) occurs in the middle and apical 1/3 areas of the root (black arrowheads). C, cementum; AB, alveolar bone; D, dentine; and PDL, periodontal ligament. The scale bars represent 500 μm (**A**, **D**, and **G**) and 100 μm (**B**, **C**, **E**, **F**, **H**, and **I**)

By post-replantation day 14, the PDL fibres were arranged regularly, with no absorption on the root surface in the normal control group (Fig. 2A-C). In the occluded group, the PDL fibres were regularly rearranged, although a few small resorption lacunae could be observed on the root surface (Fig. 2D-F). However, in the non-occluded group, deep resorption lacunae were observed on root surfaces. The PDL space was severely narrowed or even disappeared, with osteoid tissue connecting the alveolar bone to the absorption lacunae in the middle and apical third areas of the root, indicating the occurrence of RRR. Furthermore, bone-like tissues were observed in the root canal (Fig. 2G-I).

### Physiological occlusal force attenuated osteoclastogenesis of the replanted tooth at the early stage of the replantation

**Fig. 3:**
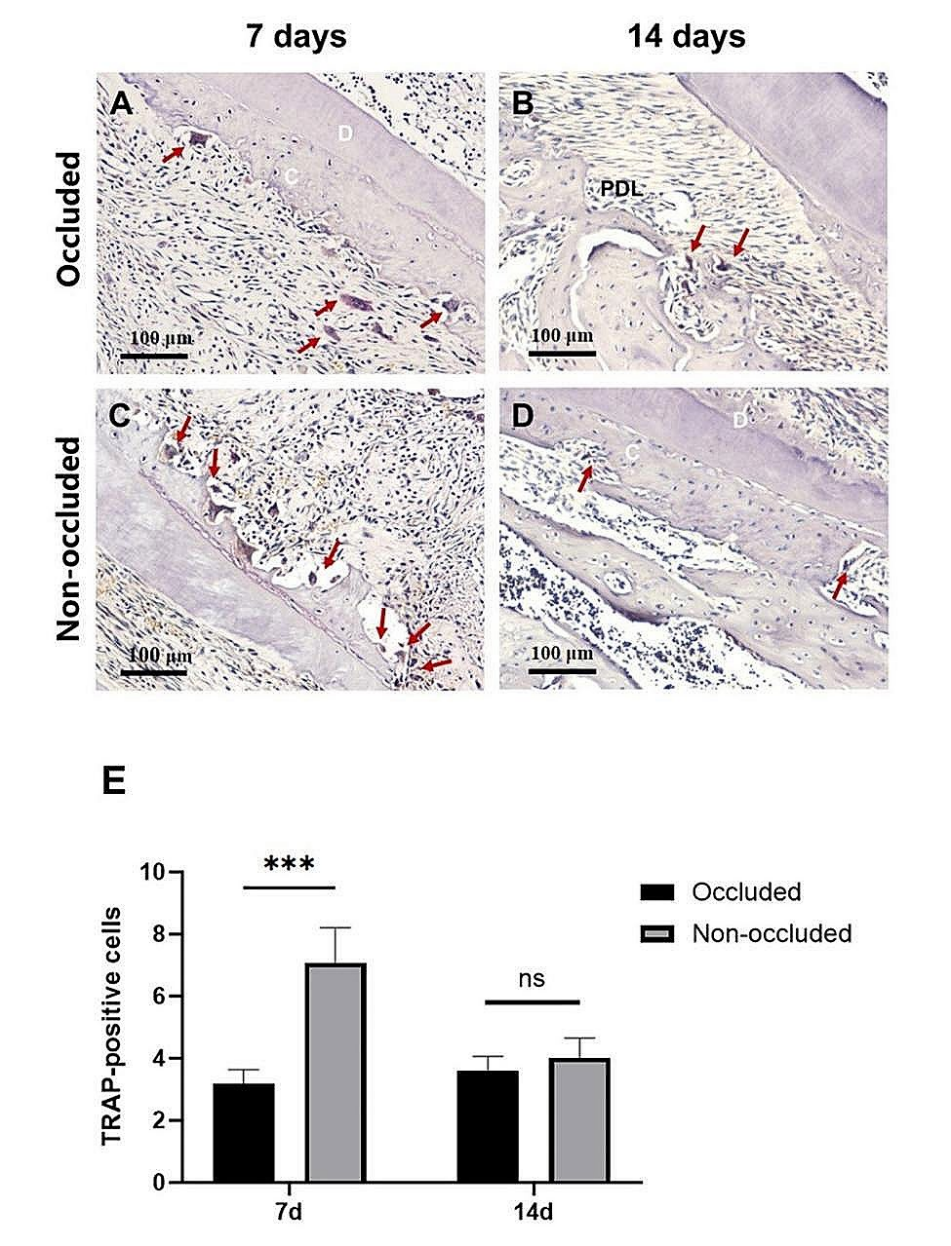
Histological observation and counting of TRAP-positive polynuclear cells after replantation by TRAP staining. A, 7 days after replantation, in the occluded group, small resorption lacunae are visible on the root surface, along with a small number of TRAP-positive polynuclear cells (red arrowheads). B, 14 days after replantation, in the occluded group, no obvious resorption lacuna is present on the root surface, except for a small number of TRAP-positive polynuclear cells near the alveolar bone (red arrowheads). C, 7 days after replantation, in the non-occluded group, large resorption lacunae are observed on the root surface, and many multiple TRAP-positive polynuclear cells are observed (red arrowheads). D, 14 days after replantation, in the non-occluded group, several resorption lacunae were observed on the root surface, with TRAP-positive polynuclear cells present in the lacunae. E, Analysis of TRAP-positive polynuclear cells on the root surface after replantation. The number of TRAP-positive polynuclear cells in the non-occluded group is significantly higher than that in the occluded group at day 7 after replantation. ***P < 0.001. ns, *P* > 0.05. C: cementum; AB: alveolar bone; D: dentine; PDL: periodontal ligament. The scale bars represent 100 μm

TRAP staining showed that the number of TRAP-positive OCs along the root surface in the non-occluded group was higher than that in the occluded group 7 days after replantation (*P* < 0.001). However, no significant difference was observed between the occluded and non-occluded groups on day 14 (Fig. 3). The statistical data for TRAP-positive cell counts across each group are presented in Table 2.

**Table 2:** TRAP-positive cell numbers: The number of TRAP-positive cells in both occluded and non-occluded groups, observed on days 7 and 14 post-replantation, determined by TRAP staining. Results are presented as the mean ± SD, with each group consisting of *n* = 6 rats, ^***^P < 0.001 vs. Occluded

Time point	Group	Sample 1	Sample 2	Sample 3	Sample 4	Sample 5	Sample 6	Mean ± SD
7 days	Occluded	3.2	2.8	3.6	3.1	3.8	2.6	3.2 ± 0.5
	Non-occluded	8.8	5.4	7.0	6.9	7.6	6.4	7.1 ± 1.1^***^
14 days	Occluded	4.4	3.4	3.4	3.5	3.0	3.8	3.6 ± 0.5
	Non-occluded	4.0	4.6	3.4	4.1	3.2	4.8	4.0 ± 0.6

### Physiological occlusal force modulated the PDL space of the replanted tooth at the late stage of the replantation

**Fig. 4:**
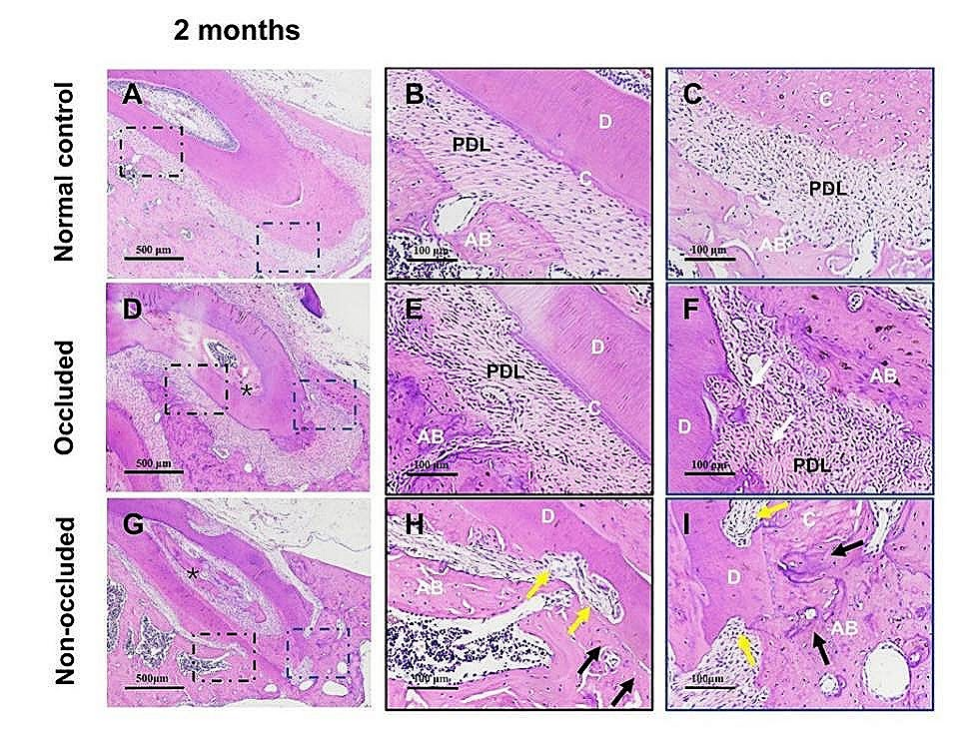
Histological observation 2 months after replantation by H&E staining. A, The PDL fibres are regularly arranged in the normal control group. B and C, High-magnification images of the middle and apical 1/3 of the root, respectively in (**A**). The root is intact with no absorption. D, The PDL fibres are rearranged regularly in the occluded group, and bone/dentine-like tissues are observed in the root canal (black stars). E and F, Local magnification of the middle and apical 1/3 of the root, respectively in (**D**). Cementum formation with reattached PDL fibres is observed in the middle and apical 1/3 areas of the root (white arrowheads). G, The PDL space almost disappears in the non-occluded group, especially in the apical 1/3 area of the root, and bone/dentine-like tissues are also observed in the root canal (black stars). H and I, Local magnification of the middle 1/3 and apical 1/3 of the root, respectively in (**G**). Severe root resorption occurred, with numerous absorption lacunae of varying sizes on the root surface (yellow arrowheads) directly connected to the alveolar bone (black arrowheads). C, cementum; AB, alveolar bone; D, dentine; and PDL, periodontal ligament. The scale bars represent 500 μm **(A**, **D**, and **G**) and 100 μm (**B**, **C**, **E**, **F**, **H**, and **I**)

H&E staining showed that the PDL fibres were arranged regularly and that the root was intact in the normal control group 2 months after replantation (Fig. 4A-C). In the occluded group, the PDL fibres were rearranged regularly between the root and alveolar bone in the middle and apical third areas of the root, indicating restorative surface absorption (Fig. 4D-F). Conversely, in the non-occluded group, the PDL space nearly disappeared, particularly in the apical third of the root, with severe RRR observed. (Fig. 4G-I). Additionally, numerous bone/dentine-like tissues were observed in the root canal in both occluded and non-occluded groups (Fig. 4D, G).

**Fig. 5:**
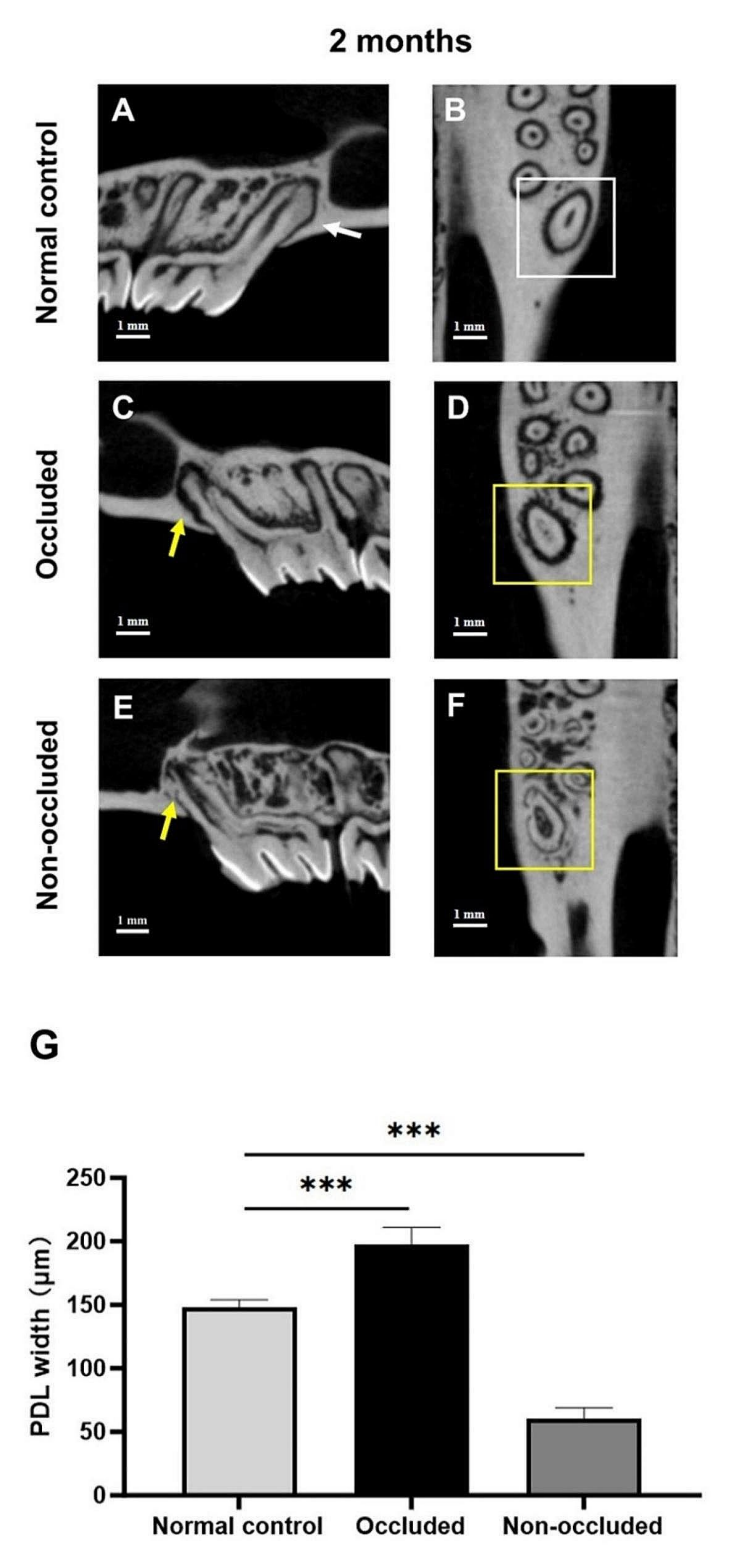
Micro-CT scanning and analysis 2 months after replantation. A and B, Sagittal and horizontal planes of the replanted tooth in the normal control group. The PDL is well-defined, with no root surface resorption (white arrowhead and frame). C and D, Sagittal and horizontal planes, respectively, of the replanted tooth in the occluded group. The width of the PDL is increased, and surface resorption in the apical 1/3 of the root is clearly visible (yellow arrowhead and frame). E and F, Sagittal and horizontal planes of the replanted tooth in the non-occluded group. The PDL almost disappears, and the root and alveolar bone are directly connected, with extensive root resorption (yellow arrowhead and frame). G, Width of the PDL 2 months after replantation among the three groups. Compared with the normal control group, the width of the PDL is significantly larger in the occluded group, and significantly smaller in the non-occluded group. ^***^P < 0.001. The scale bars represent 1 mm

At 2 months post-replantation, the micro-CT scans in the normal control group revealed a well-defined PDL space with no evidence of root surface resorption (Fig. 5A, B). In the occluded group, there was a slight increase in the PDL space width, and surface resorption in the apical 1/3 region of the root was evident, although no significant RRR was observed (Fig. 5C, D). Conversely, in the non-occluded group, the PDL space nearly vanished, establishing a direct connection between the root and alveolar bone, indicating an extensive RRR (Fig. 5E, F).

Correspondingly, in this study, RRR was observed in 2 out of 6 cases (33.3%) in the occluded group, while the non-occluded group demonstrated a higher incidence, with 5 out of 6 cases (83.3%) showing RRR. However, there was no statistical difference in the incidence rate of RRR (*P* = 0.2424). We further evaluated the severity of RRR between the two groups by analyzing the width of the PDL space 2 months post-replantation. The average width of the PDL space in each group was as follows: 148.13 ± 6.02 μm (normal control group), 197.35 ± 13.73 μm (occluded group), and 60.23 ± 8.74 μm (non-occluded group). Compared with the normal control group, the width of the PDL space significantly increased in the occluded group (*P* < 0.001) and decreased in the non-occluded group (*P* < 0.001) (Fig. 5G; Table 3).

**Table 3:** PDL width (µm): The width of PDL in the normal control, occluded, and non-occluded groups, measured 2 months post-replantation using micro-CT analysis. Results are presented as the mean ± SD, based on a sample size of *n* = 6 rats per subgroup, ^***^P < 0.001 vs. Normal control

Group	Sample 1 (µm)	Sample 2 (µm)	Sample 3 (µm)	Sample 4 (µm)	Sample 5 (µm)	Sample 6 (µm)	Mean ± SD (µm)
Normal Control	142.54	152.67	150.74	155.92	146.44	140.44	148.13 ± 6.02
Occluded	183.89	181.07	203.51	197.94	218.69	199.02	197.35 ± 13.73^***^
Non-occluded	57.47	69.92	59.71	45.98	68.97	59.33	60.23 ± 8.74^***^

## Discussion

In the present animal study, we conducted a comprehensive evaluation of periodontal healing post-replantation using H&E staining and micro-CT analyses, with a focus on the influence of occlusal forces on periodontal stability and adaptation. Our findings offer a perspective on the dynamic interplay between occlusal forces and periodontal tissue healing. Initially, on day 7 post-replantation, H&E staining revealed the most pronounced changes in the non-occluded group, particularly in the apical third, where more noticeable resorption lacunae were observed, indicating the adverse effects of the absence of occlusal force. These findings are consistent with the insights provided by Dewake et al., who explored the impact of occlusal trauma on periodontal tissues using MRI-based methods [18]. In line with these observations, Mine et al. reported that occlusal forces promoted periodontal healing and prevented dentoalveolar ankylosis in a rat model, highlighting the importance of mechanical stimuli for successful periodontal repair post-transplantation [9]. Interestingly, the occluded group exhibited minimal resorption lacunae, suggesting that occlusal force might play a role in reducing severe resorption. This observation aligns with the research conducted by Park et al., who demonstrated that the application of orthodontic force prior to tooth replantation resulted in enhanced PDL tissue proliferation, diminished root resorption, improved PDL fibre organisation, and significantly increased the mRNA levels of genes such as CXCL2, CCL4, CCL7, MMP3, PCNA, OPG, and RUNX2 at 7 days post-replantation [19].

By the 14th day post-replantation, notable divergences in healing patterns existed between occluded and non-occluded groups. In the occluded group, a restorative process was observed, characterized by the realignment of PDL fibres and the formation of new cementum layers on the roots. This tissue adaptation and repair highlight the protective effects of occlusal forces in periodontal health. Chen et al. demonstrated that occlusal forces augmented nitric oxide production in the PDL, thus substantiating the idea that mechanical stimuli foster an environment conducive to periodontal regeneration and stability [20]. Conversely, the non-occluded group presented a markedly different scenario, characterised by deep resorption lacunae and a constricted PDL space. These features are indicative of an accelerated RRR. Xu et al. also found that the absence of occlusal force contributes to the exacerbation of RRR. Taken together, physiological occlusal force may attenuate RRR in replanted teeth [21].

RRR results from irreversible PDLCs damage owing to improper tooth storage and prolonged extraoral drying. This progression is influenced by both environmental factors and cellular responses, particularly through the regulation of cytokines and the activity of OCs [22]. As the lifecycle of OCs indicates, their peak activity is concentrated within the initial phases of bone remodelling. Subsequently, osteoclast activity diminishes significantly, shifting toward bone formation, making additional TRAP staining at later stages less informative [23, 24]. Hence, in this study, TRAP staining was performed at 7- and 14-days post-replantation to capture the crucial early stages of bone remodelling. Within our research, occlusal force stimulation reduced osteoclast populations within 7 days post-replantation. By day 14, a convergence in osteoclast numbers across different groups was noted, suggesting that physiological occlusal force plays a vital role in modulating periodontal equilibrium. This shift is underscored by the differing lifecycles of OCs and osteoblasts. OCs typically undergo apoptosis within two weeks, whereas osteoblasts can remain active for up to three months [25]. Consequently, the immediate post-replantation phase is critical for osteoclast activity, with osteoblasts gaining prominence from days 7 to 14, thereby mitigating root resorption. The guidelines of the International Association of Dental Traumatology (IADT) recommend using a passive flexible splint for replanted teeth to balance stability with physiological occlusal forces and movement, to minimise the risk of RRR [4]. This recommendation for a 14-day splinting period is based on evidence showing that more than 60% of the mechanical properties of an injured PDL are restored within this critical timeframe [13]. Our study’s 14-day histological findings support these guidelines, revealing that physiological occlusal forces during this period significantly enhance PDL organisation and reduce osteoclast activity, thereby improving the prognosis of replanted teeth. Conversely, the absence of physiological occlusal force is linked to upregulated osteosclerotic protein expression in osteoblasts and reduced activation of Gli1^+^ PDL stem cells, contributing to decreased periodontal tissue [26]. Additionally, TNF-α is pivotal in osteoclast genesis within the PDL under mechanical loading, as evidenced in TNF-receptor-deficient mice, which display reduced orthodontic tooth movement and fewer OCs [27], elucidating the intricate relationship between mechanical forces and bone remodelling at the cellular level. Nonetheless, the exact molecular pathways through which these forces influence RRR in replanted teeth have yet to be fully elucidated.

Two months post-replantation, our study unveiled a complex panorama of periodontal healing shaped by the interplay of mechanical forces. In the normal control group, the H&E staining revealed a well-preserved PDL structure with regular fibre arrangement, corroborated by micro-CT scans that exhibited a well-defined PDL space. This concordance reinforces the integral role of a stable mechanical environment in maintaining periodontal health, as evidenced by the absence of root surface resorption. Conversely, the occluded group, characterised by a slight increase in PDL space as per micro-CT analysis, showed a regular rearrangement of PDL fibres through H&E staining, indicative of a dynamic healing process. This observation aligns with the understanding that controlled mechanical forces can foster a balanced environment conducive to tissue repair and remodelling [28, 29].

Characterised by the fusion of root and bone, RRR manifests as a continuous radiographic image without distinct separation [30]. Computed tomography provides superior accuracy in detecting RRR [31], which benefits earlier identification compared with conventional X-ray imaging. In this animal study, micro-CT observations revealed a 33.3% occurrence of RRR in the occluded group, and a higher rate of 83.3% in the non-occluded group, showing an increased trend of RRR. Further evaluation of average width of the PDL space demonstrated greater severity of RRR in the non-occluded group. These findings suggest a potential protective role of physiological occlusal forces against RRR; nevertheless, larger sample sizes are needed to establish this relationship more definitively. Supporting this notion further, researchers highlight the advantages of occlusal forces in fostering a dynamic healing process and averting adverse outcomes such as ankylosis. They suggest the crucial equilibrium between resorption and regeneration in periodontal healing and adaptation [9, 20]. The presence of surface resorption in the apical third, as detected by micro-CT, suggests a nuanced balance between resorption and regeneration, a theme resonant in the realm of periodontal healing and adaptation. The severe constriction of the PDL space and the onset of extensive RRR, establishing a direct connection between the root and alveolar bone, illustrate the deleterious impact of the absence of occlusal forces on periodontal structure and integrity. The presence of bone/dentine-like tissues within the root canal, as observed by H&E staining, suggests a complex process of tissue differentiation under varying mechanical conditions. This aspect of tissue differentiation under stress aligns with the themes explored by Dewake et al., who proposed novel imaging techniques for evaluating mechanical impact on periodontal tissues [18].

Our study provides valuable insights into the role of physiological occlusal forces in enhancing periodontal healing in replanted teeth, emphasizing their importance in mitigating RRR. However, it is crucial to acknowledge the limitations inherent in our research methodology. While the rat model used offers a controlled environment for studying the effects of occlusal forces, it may not fully capture the complexities of human periodontal healing processes. Future research should focus on understanding the differences between animal models and human periodontal responses to better predict and interpret clinical outcomes in humans. Additionally, investigating the long-term effects of occlusal forces on periodontal healing is essential for a more comprehensive understanding of the healing process and for improving treatment strategies, particularly during the later stages of healing. This study underscores the necessity of a balanced approach for the clinical management of replanted teeth, highlighting the complex interplay between physiological occlusal forces and periodontal tissue healing.

## Conclusions

In this study, we present preliminary findings indicating a significant role of physiological occlusal forces in curbing osteoclastogenesis during the initial phases of tooth replantation. This process appears to be instrumental in reducing RRR and promoting periodontal healing. Our results reveal a new dimension in understanding the relevance of occlusal forces within the realm of dental trauma care.

## Data Availability

The authors confirm that the data supporting the findings of this study are available within the article.

## Abbreviations

PDL: Periodontal Ligament
PDLCs: Periodontal Ligament cells
RRR: Replacement Root Resorption
OCs: Osteoclasts
H&E: Haematoxylin-Eosin
TRAP: Tartrate-Resistant acid Phosphatase
micro-CT: Micro-Computed Tomography
